# Comparison of a Chinese Herbal Medicine (CCH1) and Lactulose as First-Line Treatment of Constipation in Long-Term Care: A Randomized, Double-Blind, Double-Dummy, and Placebo-Controlled Trial

**DOI:** 10.1155/2012/923190

**Published:** 2012-03-18

**Authors:** Chien-Hsun Huang, Jui-Shan Lin, Tsai-Chung Li, Shih-Chang Lee, Hsiu Po Wang, Hung-Chi Lue, Yi-Chang Su

**Affiliations:** ^1^Department of Community and Family Medicine, National Taiwan University Hospital Yun-Lin Branch, Yun-Lin, Taiwan; ^2^Graduate Institute of Chinese Medicine, College of Chinese Medicine, China Medical University, Taichung, Taiwan; ^3^School of Chinese Medicine, College of Chinese Medicine, China Medical University, Taichung, Taiwan; ^4^Graduate Institute of Biostatistics, China Medical University, Taichung, Taiwan; ^5^Division of Gastroenterology, Department of Internal Medicine, National Taiwan University Hospital, Taipei, Taiwan; ^6^Department of Pediatrics, National Taiwan University Hospital, Taipei, Taiwan

## Abstract

Many institutionalized patients and their healthcare providers are dissatisfied with current laxative therapy. This study compared therapeutic efficacy, safety, and laxative cost of an herbal formula (CCH1) and lactulose for long stay patients with constipation. In this double-blind, double-dummy, and placebo-controlled trial, we randomized 93 residents with chronic constipation from two long-term care facilities in Taiwan to receive either CCH1 with lactulose placebo or CCH1 placebo with lactulose for 8 weeks, then followed up for 4 weeks without study medication. Both treatments were effective and well tolerated for patients, but CCH1 produced more spontaneous bowel movements, less rectal treatments, less amount of rescue laxative, and lower laxative cost than lactulose during treatment. No significant differences were found in stool consistency, stool amount, global assessment, and safety concerns. In conclusion, our results suggest that CCH1 may have better efficacy and could be used as an alternative option to lactulose in the treatment of constipation in long-term care.

## 1. Introduction

Constipation is often dismissed as a trivial medical concern, it can be associated with mild-to-extreme distressing and potentially dangerous complications [1]. Almost half of all patients older than 65 reported constipation or the regular use of laxatives [2]. In nursing facilities, 72.7% of the patients had at least one precipitating comorbid condition for constipation, and 77.6% of the residents were receiving at least one medication that may contributed to the occurrence of constipation [3]. Accordingly, the prevalence of constipation is higher in long-term care with up to 74% of nursing home residents using daily laxatives [4]. In fact, constipation often leads to deterioration in health-related quality of life [5] and also increases time and economic cost of constipation care in long stay [6].

Dissatisfied with conventional therapy, there is a trend that herbal medicine use increased substantially in the United States, and over one in three Americans used complementary and alternative medicine (CAM) in the past year [7]. In Taiwan, more than 95% of citizens have medical insurance under the national health insurance (NHI) system. The use of Chinese medicine is also reimbursed by the NHI, and all qualified residents in Taiwan are free to choose either Western medicine or traditional Chinese medicine (TCM) [8]. A national survey in Taiwan reported 152,564 subjects who visited TCM clinics only for constipation during 2004 [9]. Constipation is also of the top three main diagnoses in a Traditional Medicine Center of a general hospital in Taiwan [10].

In fact, TCM has been the primary system of medicine in Asia for thousands of years. According to the theories of TCM, constipation can be generally divided into excessive and deficient syndromes (*ying, yang, Qi* or *blood* deficiency), based on the underlying etiology [11, 12]. With regard to the syndrome of *yang* deficiency, it is a pathological state resulting from deficiency of “*yang*” energy with reduction in its warming and activating power that leads to diminished functions (including GI system), decreased metabolic activities, intolerance to cold, and so on [13, 14]. “*Wen-Pi Tang”*, derived from a classic TCM book named *Qian Jin Fang *(chapter 15) in the Tang Dynasty (A.D. 652), is a famous formula that has been commonly used throughout Asia for constipation of the “*yang* deficiency” type [15, 16]. It was composed of five herbs (*Ren Shen, Gan Jiang, Zhi Fu Zi, Da Huang, and Gan Cao*) [15, 16]. Through the combined action of these herbs, *Da Huang* served as purgative drug with the assistant of the rest of four herbs formulated to strengthen the energy of *yang *[14, 16]. From the viewpoints of TCM, the majority of patients with constipation of *yang* deficiency are the frail older people or chronically ill patients [11, 15]. Since many residents in nursing homes are the dependently older people with comorbidities, most of their constipation could be attributed to *yang* deficiency in some degree. Therefore, our previous randomized placebo-controlled trail has demonstrated CCH1 (Table 1), an herbal formula modified from “*Wen-Pi Tang”*, to be safe and effective on the treatment of constipation for the population in nursing homes [17].

Despite the enthusiasm for CAM, the high-quality comparative study of herbal supplements and western medicine for constipation is limited [18, 19]. From the review of literature, osmotic laxatives are effective in older adults and well tolerated [1, 20]. in the UK, the most commonly used of osmotic agents is lactulose [21], which is also popularly used in Taiwan. therefore, we aimed to compare the efficacy, safety, and cost of CCH1 and lactulose for treating constipation in Long-Term Care. 

## 2. Materials and Methods

### 2.1. Participants

The participants were the residents of two nursing homes in mid Taiwan. They received usual medical care from one surrounding teaching hospital in the same community. The study was conducted from August 2008 through April 2010. The inclusion and exclusion criteria are shown in Table 2.

The study was performed in compliance with the principles of the Declaration of Helsinki and following “good clinical practice” guidelines. The study protocol was approved by the Research Ethics Committee of National Taiwan University Hospital before conducted.

### 2.2. Study Medication

The herbal preparation was obtained from *Sun Ten* international pharmaceutical company, a qualified manufacturer of concentrated herbal extracts with good manufacturing practice. The herbs were authenticated at the Brion Research Institute in Taiwan on the basis of standards specified in the *Taiwan Pharmacopoeia of Chinese Medicine* (2004 edition). Contamination screening for heavy metals, pesticides, and aflatonix was performed to ensure safety for human consumption. For quality control and standardization of CCH1, herbal preparation was performed in a single batch to ensure consistency of quality. The indicator ingredient of CCH1 was analyzed by high-performance liquid chromatography (HPLC). After decoction and extraction, herbal preparation was concentrated into the powdered form and packed in sealed opaque aluminum foil bags.

With the reference of a double-blind, and placebo-controlled trial of an herbal formula [22], the placebo of CCH1 was made of similarly colored starch, which was packed in an identical package and manufactured by the same company as CCH1. With the other reference of a study for elderly constipated patients, 50% of glucose water was used as placebo-controlled of lactulose [23]. For similar viscosity and taste with lactulose, the placebo of lactulose in our study was manufactured by the same company as lactulose syrup, which was composed of 500 mg glucose, 2 mg xanthum gum (a polysaccharide used as a food additive to increase the viscosity of a liquid), 0.01 mg Sunset yellow FCF (an artificial coloring allowed to be added to food), and 497.99 mg purified water in each gram content. For the ingredients of placebo in this study, no known laxative effects or drug interaction with study medication existed.

### 2.3. Study Design

This double-blind, double-dummy, and placebo-controlled study had two parallel groups with a total treatment phase of 8 weeks. After 8 weeks of treatment, patients were followed up for one additional month without study medication (CCH1 or lactulose) and placebo except for rescue laxative of magnesium oxide (MgO). To obtain baseline information, all patients had a run-in period of 2 weeks without laxatives but only concurrent use of CCH1 placebo and lactulose placebo for ethical concerns and test for compliance before randomization.

With the reference of the national guidelines of the Registered Nurses Association [24], daily stool diaries were kept by the certified nurse assistants from the beginning of the run-in period to the completion of the study, including stool frequency, stool consistency, stool amount, and the use of rectal treatment (RT; enema, suppository use, or digital maneuver). Stool consistency was recorded by Bristol Stool Form Scale, ranging from 1 (separate hard lumps, like nuts) to 7 (watery, no solid pieces) [25]. Stool amount was classified into three categories (small, moderate, and large) according to the national guidelines [24]. In addition, a bowel routine protocol was set: if there was no bowel movement (BM) by day 3, the caregiver gave a suppository of bisacodyl or glycerin ball on day 3; if there was no BM after 8 hours of suppository insertion, enema was given. Meanwhile, when necessary, the caregiver could apply a digital maneuver to manually remove the hard stool or stimulate the rectum. Therefore, the bowel performance kept in the stool diary could imply the severity of constipation clinically.

Considering of the real situation in long-term care, physicians usually prescribe laxatives with different dosage according to the severity of constipation based on the stool diary. The participants were classified into three groups for initial dosage of study medication by enema frequency and weekly spontaneous bowel movement (SBM) during the run-in period. Having enema more than once a week was regarded as group A. Residents who received enema once a week or had less than three SBMs per week belonged to the group B. The frequency of SBM three to seven times a week was referred to as group C. Participants averaging more than one SBM per day during the run-in period were excluded.

Residents were randomized to the study medication according to a computer-generated randomization schedule. Randomization was stratified based on the severity of constipation and Barthel's index [26], a scale used to measure functional performance in basic activities of daily living. Allocation concealment was performed by enclosing assignments in sequentially numbered, identical, and sealed two envelopes according to the allocation sequence in every stratified group. The packaging of study medication and placebo were indistinguishable, and they were dispensed after randomization by an independent research assistant in a separate office. All persons involved in the conduct and management of the study were blinded to individual patient treatment during the study.

### 2.4. Interventions

The experimental group received an initial dose of 4.5/3.0/1.5 gm CCH1 powder with 45/30/15 mL lactulose placebo per day for groups A/B/C, respectively. The active comparator group was given an initial dose of 45/30/15 mL lactulose with 4.5/3.0/1.5 gm CCH1 placebo per day for groups A/B/C, respectively. If optimal bowel performance [27] (3 SBMs/wk to 3 SBMs/day without any rectal treatment) was not reached, study medication were titrated by weekly increments of “1.5 gm of CCH1 and 15 mL of lactulose placebo for experimental group” or “15 mL of lactulose and 1.5 gm of CCH1 placebo for comparator group” until the maximal daily dose of 6.0 gm for CCH1/CCH1 placebo or 60 mL for lactulose/lactulose placebo. Daily dosage over 3.0 gm of CCH1/CCH1 placebo or over 30 mL of lactulose/lactulose placebo was prescribed by divided dose.

During the whole study period of 12 weeks, MgO was the only rescue oral laxative allowed to be used. In the treatment phase of weeks 1–8, if optimal bowel performance was not reached under the maximal dose of study medication, MgO was added by the blinded primary physician and was titrated every week (increased by up to 750 mg/day once a week, maximum dose of 2.0 g/day).

### 2.5. Assessment

Participants were visited every week by one primary physician with licenses for both conventional medicine and TCM during the entire study period of 12 weeks. SBM was defined as stool passage without digital maneuver and without the use of suppository or enema on the same day. The cost calculations of lactulose and MgO were derived from the reimbursed payment of the national health insurance in Taiwan. The cost assigned to the investigational product (CCH1) was derived from current wholesale distributor pricing. Using a 5-point Likert scale, the global assessment of efficacy was evaluated by patients themselves or their principal caregivers if their cognition was impaired.

### 2.6. Efficacy

The frequency of SBM during weeks 1–4, 5–8, and 9–12 was measured as primary efficacy. Secondary end points included (1) frequency of RT during weeks 1–4, 5–8, and 9–12; (2) weekly amount of rescue laxative use during weeks 1–4, 5–8, and 9–12; (3) weeky laxative cost during treatment phase of weeks 1–4 and 5–8; (4) average score of Bristol Scale during weeks 1–4, 5–8, and 9–12; (5) mean proportion of different stool amount (small, moderate, and large) per week during weeks 1–4, 5–8, and 9–12; (6) global assessment of efficacy at the end of treatment phase; (7) cumulative incidence of adverse events during the entire study period.

### 2.7. Safety

Safety evaluations included adverse events and serious adverse events reporting, vital signs, physical examination findings, and laboratory results. Adverse events were monitored with a comprehensive symptom questionnaire as well as clinical laboratory testing—including complete blood count, plasma sugar, aspartate aminotransferase, alanine aminotransferase, total bilirubin, alkaline phosphatase, urea nitrogen, creatinine, albumin, thyroid-stimulating hormone, free thyroxine, sodium, potassium, calcium, phosphate, magnesium, uric acid, triglyceride, and total cholesterol—carried out before and after treatments in a qualified laboratory.

### 2.8. Statistical Analysis

An intention to treat analysis was conducted on all patients randomized for therapy. All patients who had taken at least one dose of study medication after randomization were included in the safety analysis. A last observation carried forward analysis was conducted for any missing data of primary or secondary outcomes except global assessment of efficacy and adverse events. Since primary efficacy and some secondary end points were to measure the outcomes at different time periods, the Bonferroni adjustment for multiple comparisons was used to assess the statistical significance of these multiple tests. Statistical analysis was performed using SPSS software version 13 (SPSS, Chicago, IL, USA). Numerical data were compared using the Student's *t*-test or Mann-Whitney *U* test. The paired *t*-test was used to compare individual bowel performance before and after treatment. A *χ* ^2^ test or Fisher exact test where appropriate were used to compare the number of patients with adverse events, global assessment of efficacy, and categorical data of patients' characteristics in baseline period between two groups. These comparisons were made at a 2-sided *α* level of 0.05.

### 2.9. Sample Size Considerations

Based on previous clinical trial of CCH1 and the methodological recommendations of a systematic review [17, 21], the standard deviation of the mean weekly SBM was estimated to be 2.0. A total sample size of about 93 patients would be required to detect a mean difference between treatments of 1.5 bowel movements per week under the assumptions of 90% power, a 2-sided *α* value of 0.05 and allowance for a 20% drop-out rate.

## 3. Results

### 3.1. Participants, Study Conduct, and Completion

**Figure 1:**
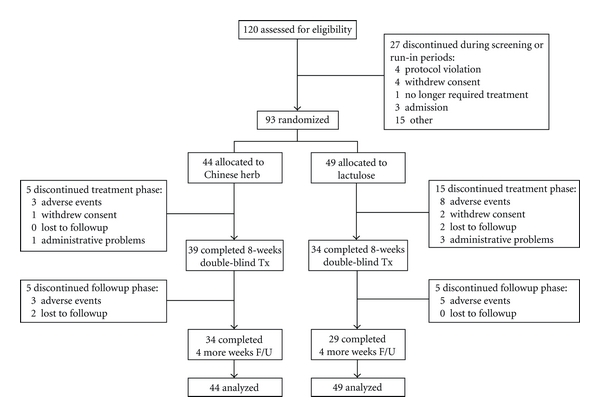
Participant flowchart depicting the randomization, treatment, and followup in CCH1 and lactulose groups.

A total of 120 participants who had signed the informed consent were screened for eligibility; 93 (77.5%) met the inclusion criteria and were randomized. The reasons for exclusion are shown in Figure 1. Over 80% of the 93 enrolled patients were older than 60 years with mean age of 73.5 and 54.8% were female. Patients' baseline characteristics were similar between the two treatment groups (all *P* < 0.05; Table 3). A total of 20 (21.5%) patients dropped out of the trial during the treatment phase (weeks 1–8): 5 (11.4%) in the CCH1 group and 15 (30.6%) in the lactulose group (*P* = 0.041). Among the 15 dropouts in lactulose group, 8 people had severe adverse events and hospitalized (pneumonia: 2, acute gastroenteritis: 2, upper gastrointestinal bleeding: 1, urinary tract infection: 1, ileus: 1, and cellulitis: 1); 2 persons withdrew their consents and one of them complained lactulose syrup was too sweet; 2 residents were lost to followup because transferred to other facilities; one person was withdrawn on the ethical concerns for refractory constipation under the maximum dosage of treatment medication and rescue laxative; another 2 persons were dropped from the study for poor compliance (Figure 1). Thirty-four (77.3%) patients in the CCH1 group and 29 (59.2%) patients in the lactulose group completed the study (*P* = 0.077). There was no significant different on demographics between drop outs and nondrop outs.

### 3.2. Primary Efficacy Analysis

During treatment phase, mean daily dose of study medication was 3.8 (standard deviation 1.4) gram CCH1 in experimental group and 38 (SD 14) mL lactulose in comparator group. No significant differences were found in the frequency of SBM in baseline period between the two groups (mean difference 0.1 [95% CI −0.9 to 1.0]). Mean numbers of weekly SBM in CCH1 group were greater than those in lactulose group during 8-week treatment phase (6.8 versus 5.0, difference 1.8 [0.7 to 3.0]; *P* = 0.001); greatest difference was during weeks 1–4 (6.9 versus 4.5, difference 2.4 [1.2 to 3.6]; *P* < 0.001). However, in the follow-up phase of weeks 9–12, the frequency of SBM in CCH1 group was less than that in lactulose group (3.7 versus 5.2; *P* = 0.084),but still greater than its baseline period (3.7 versus 2.7; *P* = 0.005) (Table 4).

### 3.3. Secondary End Points

Smaller mean numbers of RT/wk were observed in the CCH1 group compared with the lactulose group during weeks 1–4 (0.5 versus 0.9, difference −0.4 [−0.7 to −0.1]) and weeks 5–8 (0.3 versus 0.6, difference −0.3 [−0.5 to −0.1]). Regarding the need for a rescue laxative (MgO), the mean numbers of tablets of MgO/wk were less for the patients who were given CCH1 than for those who received lactulose during the entire treatment phase; the greatest difference was during weeks 5–8 (1.7 versus 10.4, difference −8,7 [−13.4 to −3.9]). However, no significant differences were found in the frequency of RT/wk (1.0 versus 0.8) or in the amount of MgO/wk (19.1 versus 20.0) between the two groups during follow-up phase of weeks 9–12 (Table 4).

Compared with the lactulose group, the laxative cost (USD/wk) in CCH1 group was less during weeks 1–4 (1.4 versus 4.7, difference −3.3 [−3.8 to −2.8]) and weeks 5–8 (1.5 versus 5.0, difference −3.5 [−4.2 to −2.8]) (Table 4). No significant differences were found in stool consistency or stool amount between the two groups at all time points (Table 5). The 73 (78.5%) patients who completed the treatment phase were investigated for global assessment of efficacy at week 8, 84.6% of CCH1 patients had marked or slightly improvement versus 79.4% in the lactulos group (*P* = 0.646) (Table 6). 

### 3.4. Safety and Compliance

All patients in both groups had good compliance of around 97% with study medication except for three patients with 70–80% (Table 7). Six (13.6%) in CCH1 and 12 (24.5%) in lactulose patients discontinued the study because of severe adverse events (SAEs) (*P* = 0.203; Table 8). No significant differences were found between the two groups in the incidence of any one of the common AEs or SAEs (Tables 8 and 9).

## 4. Discussion

In this randomized, double-blind, double-dummy, and placebo-controlled trial, we found that both CCH1 and lactulose are effective and well tolerated in long stay patients with constipation. However, the regimen of CCH1 produced more frequent SBM, less frequent RT, lower amount of rescue laxative, and lower laxative cost than lactulose during treatment of 8 weeks.

In TCM, “syndromes” are the foundations for therapeutic principles [11]. However, to date there is no international consensus or consistently applied classification or criteria of syndromes for many diseases and symptoms such as constipation [28]. And the process of syndrome identification performed by TCM doctors is very subjective and usually lacks interrater consistency because it is highly dependent on the TCM doctors' personal skills including evaluating the nature of patient's pulse and appearance of the tongue [28]. That is why clinical trials of TCM are often questionable about whether the study results are reproducible for other research teams or generalizable to the Western patients [29]. By contrast, in our study, the majority of study population in long-term care was estimated to be the pattern of *yang *deficiency in terms of TCM theories [15]. Therefore, without the need to or help of TCM practitioners, this study is easier to be replicated or translated into routine care of constipation in conventional medicine. However, the relationship of therapeutic effect of CCH1 with the degree of *yang* deficiency in long stay residents needs to be investigated.

From literature review, there is no consistently applied definition of constipation in the elderly [30]. Definition such as the Rome III criteria, however, may not encompass the perceptions of all patients with constipation [20]. Patients' definitions are often qualitative and include stool consistency, difficulty with passage, requirement of manual, medicinal, or other maneuvers to evacuate feces [31, 32]. In addition, constipation is often multifactorial in origin (including primary and secondary causes) with mixed types of pathophysiology in long-term care [33]. And the long stay residents in our study had potentially poor cognitive function or expression ability. Therefore, we added the other two inclusion criteria of constipation (“receiving enema, suppository or digital maneuver” and “laxative use”) to Rome III criteria, which are composed of several “subjective” complaints for “functional” constipation.

For the classification of constipation severity, the residents who had bowel frequency of 3–7 times a week during run-in period were referred to as group C (mild constipation). Although physicians often use the quantitative definition of <3 bowel movements per week to describe constipation, there is lack of agreement on the definition of constipation regarding what patients perceive and what physicians traditionally see as constipation [1]. Furthermore, placebo was given in the run-in period, which is associated with high rates of resolution in a functional bowel disorder [34]. And variation in symptoms over time is the nature of functional disorders, which may contribute to the “temporary” improvement of constipation during run-in period. Therefore, group C patients are not excluded as the study design of a previous clinical trial [17].

Moreover, since high percentage of residents in long-term care had precipitants of constipation including medical conditions and related medications [3], exclusion of these patients in clinical trial of constipation in long stay is neither practical nor ethical. Therefore, we kept the patients and investigated their medical illness. After randomization, major diseases in the patients were equally distributed between both groups as described in Table 3. In addition, for ethical reasons, all medications except laxatives were left for participants as prescribed before enrolled. Although there was no detailed record or analysis about every category of medication that patients used during the study, it is reasonable to infer that disease-related medications were randomly distributed and balanced between the two groups as that of medical illness. However, further study with explicit survey of related medications is still essential.

For the primary outcome of SBMs, the mean numbers of weekly SBM in CCH1 and lactulose groups were 6.8 ± 2.2 and 5.0 ± 2.9, respectively, during 8-week treatment phase. The pooled standard deviation was calculated to be 2.6, which is slightly larger than the assumed one (SD = 2.0) used in the sample size calculation. However, the observed effect size here (1.8) is also larger than the expected value of 1.5 bowel movements, making the standardized effect size of 0.7 (1.8/2.6) close to the expected one of 0.75 (1.5/2.0). Therefore, after calculation by the software package for sample size and power estimation PASS (Power Analysis and Sample Size, Kaysville, UT, USA), we still have the power of 0.91 to tell the difference of SBM frequency between the two groups. With regard to the negative results of secondary outcomes, although it could be attributed to the lower power (0.1–0.5) to identify the differences between groups, some of these effect sizes observed here were actually small and clinically insignificant. Further study with larger sample size may be needed to identify the significance of the outcome measures with small effect size.

Although the prespecified LOCF was applied as our analytic method, it could not be the most appropriate way to deal with the missing data. It is of particular concern that the dropout rate was different between the two groups during study period. Because more dropouts in the lactulose group came from “adverse events” and all of them were hospitalized for “severe adverse events”, it is reasonable to infer that these dropout patients' constipation would become worse after admission. Our estimation in the outcome variables at endpoint for the lactulose group would result in a better status under the imputation method of LOCF because of its assumption that the response remains constant at the last observed value. Under this condition, the efficacy of CCH1 group would be underestimated. Thus, the biased results in the effect are toward the null, a lesser threat to validity. In addition, LOCF was the most common method that was used in the literature [35], and mixed models for handling the missing data do not work well due to our small size of sample [36].

According to a recent systematic review [18] of 137 studies on the efficacy of TCM for the management of constipation, only 21 clinical trials were high of quality with participants' mean age of 50 years and TCM intervention of 20.6 days. Compared with them, our study has older participants of 73 years and longer intervention of 2 months. Among the 21 clinical trials of high quality, there were eight studies to compare TCM with Western medicine, which were all published in Chinese, and the results showed that Chinese herbal medicine were more effective than conventional medicines (cisapride, mosapride, or phenolphthalein). Compared with all the active controls selected in the eight studies, only lactulose used in our study had good supporting evidence for the treatment of chronic constipation [19, 37]. Therefore, CCH1 is the first Chinese herbal Medicine, which results of this study suggesting a better efficacy than lactulose as a treatment option for constipation in long-term care.

The double-blind, randomized, and placebo-controlled trial is the gold standard method to test the efficacy of a new treatment [38]. However, the herbal supplements with quality placebo-controlled trials to evaluate their effect on constipation were limited from the literature review [18, 19]. Furthermore, for the long-term care setting, little evidence is available from high-quality randomized controlled trials [39]. The tincture of jalapa, derived from a tropical plant in Brazil, is shown to be effective in the acute treatment of functional constipation [40]. Yun-chang capsule, a Chinese herbal formula, is efficacious and safe on the treatment of functional constipation for patients with deficient syndrome of *Qi *and *yin *[41]. Hemp Seed Pill, a TCM proprietary medicine, is safe and effective for alleviating functional constipation for subjects in excessive syndrome [35]. The study population in the three herbal trials was all targeted to the ambulatory outpatients with mean age of 33–42 years [35, 40, 41]. For the residents in long-term care, Smooth Move herbal tea, when added to the standard treatment with usual laxatives, increased bowel movements compared to the addition of a placebo tea [42]. In comparison with Smooth Move as a complementary agent for current laxatives, CCH1 in our study was documented to be a good alternative laxative to lactulose as first-line treatment for long stay patients with constipation.

Compared with lactulose used in our study, although polyethylene glycol (PEG) has not been studied in trials of exclusively geriatric populations [43], a Cochrane review indicates that PEG is better than lactulose in outcomes of stool frequency and stool consistency [44]. However, a drug class review conducted by the FDA noted that high doses of PEG may produce diarrhea and excessive stool frequency, particularly in elderly nursing home patients [45]. Lactulose and the other nonabsorbable sugar, sorbitol, appeared to be equally efficacious, but sorbitol is recommended as a cost-effective alternative to lactulose for the treatment of constipation in the elderly [46]. Lactitol, a widely prescribed osmotic laxative in India, is also suggested to be preferred over lactulose for adults with chronic constipation because of its comparable efficacy, better palatability and lesser incidence of adverse events [47]. In addition, a combination of senna plus fibre is more cost-effective and efficient than lactulose in treating constipation in long stay elderly patients [48]. Therefore, further studies to compare the efficacy, safety, and cost-effectiveness of CCH1 with other osmotic agents, including PEG, sorbitol, lactitol, or combination laxatives for the treatment of constipation in long-term care are encouraged.

There are some strengths in our study. The clinical trial of head-to-head comparison of CCH1 and lactulose was conducted with a strict and accepted methodological protocol. Compliance was around 97%, which is excellent for a trial in older subjects. Furthermore, unlike most randomized controlled trials for constipation conducted in ideal conditions [39] or in the population of ambulatory community-dwelling adults [19, 49], this study was conducted in the real situation without interference with the usual care and bowel routine for the long-term care residents, who have many nonmodifiable risk factors such as polypharmacy and coexistent medical conditions. In addition, the clinical trial fully adheres to the rationale of medical prescription in conventional medicine, which usually prescribing initial dosage of laxatives based on the severity of constipation, then titrating according to drug response.

This study has some limitations or weakness. First, test of success of blinding was not performed in the study. Second, because the residents in long-term care had potentially impaired cognitive function or poor expression ability due to old age with frailty, multiple medication, and chronic illness, objective data from medical records and stool diaries were used for outcome measurements rather than the subjective assessment of symptom score or quality of life. Third, the main population in the study was older adults living in long-term care. Therefore, the effect of CCH1 for young residents in long stay or the elderly in the community remains to be investigated. Fourth, the medical cost in the study was limited to the pharmacological cost of national health insurance in Taiwan, which is not generalizable to different countries or different health insurance systems. Moreover, the true cost of managing constipation is impacted on by a broad range of resources and not only laxative acquisition costs [50]. Therefore, further clinical trials may be necessary to compare the economic impact of CCH1 with current laxatives, including time, health providers' visits, caregivers' burden, and labor cost. Fifth, for the considerations of ethical concerns and real situation in clinical practice, when study medication was titrated to maximal dose, our study design allowed the primary physician to add and adjust the dosage of rescue laxative every week until optimal bowel performance was observed. Therefore, the laxative effect in the study was probably attributed to the combined effect of study medication and MgO. In addition, because CCH1 had better laxative effect than lactulose on SBM during treatment, the weekly amount of MgO was less in CCH1 group than that used in the lactulose group during treatment phase and the beginning of follow-up phase (4.2 versus 14.1 at week 9, *P* = 0.002). Therefore, we did not have enough evidence to compare the maintenance effect of treatment medication by the study design.

## 5. Conclusions

In conclusion, both CCH1 and lactulose were efficacious and well tolerated but CCH1 significantly increased spontaneous bowel movements and concurrently reduced the need of rectal treatments and additional laxative at lower laxative cost during treatment. Although the maintenance effect of CCH1 was uncertain, it still suggested good value for new drug development. A properly designed large-scale or multicenter clinical trial with longitudinal dada analysis is essential. Further comparative study with other laxatives is also encouraged, particularly with regard to concerns about quality of life and subjective outcomes.

## Figures and Tables

**Table 1:** The composition of CCH1.*

Manderin pronunciation	Botanical name	Gram
Ren Shen	*Panax ginseng* C. A. Meyer	0.8
Gan Jiang	*Zingiber officinale* Rosc.	0.8
Gan Cao	*Glycyrrhiza uralensis* Fisch.	0.8
Bai Zhu	*Atractylodes macrocephala* Koide.	0.8
Zhi Fu Zi	*Aconitum carmichaeli* Debx.	0.8
Da Huang	*Rheum tanguticum *Maxim. ex Balf.	1.2

*Every 3.0 g extract powder are prepared from the above raw herbs.

**Table 2:** Inclusion and exclusion criteria.

Inclusion criteria	
Age, 20 years and older	
Colonic evaluation (colonoscopy or barium enema) within the previous 5 years or regular followup at gastrointestinal clinic	
Chronic constipation confirmed by at least one of the following three criteria:	
Meeting Rome III criteria of constipation	
Receiving enema, suppository, or digital maneuver at least once a week in the past three months	
Laxative use in more than half time of the last three months	

Exclusion criteria	

Known cause of colorectal obstruction or structural lesions (e.g., intestinal neoplasm, anal abscess, anal fistula, anal fissure, rectocele, and megacolon)	
Inflammatory bowel disease	
Irritable bowel syndrome	
Hypothyroidism	
Spinal cord injury	
Muscular dystrophy	
Known severe hepatic or renal insufficiency (e.g., liver cirrhosis or receiving hemodialysis)	
Unknown cause of gastrointestinal bleeding or acute infection	
Exposure to any other investigational drug within 30 days prior to enrollment	
History of allergy to the composition of the study medication	
New onset or unstable psychiatric disorders	
Pregnancy or breastfeeding	
History of alcohol or drug abuse	
Any condition associated with poor compliance with medical treatment	

**Table 3:** Characteristics of patients in baseline period.

Variables		Group, number (%)		*P* value
		CCH1	Lactulose	
Gender				
Female		26 (59.1)	25 (51.0)	0.43
Male		18 (40.9)	24 (49.0)	
Age (years)				
41–60		9 (20.5)	9 (18.4)	0.59
61–80		17 (38.6)	24 (49.0)	
>80		18 (40.9)	16 (32.7)	
Mean (SD)		73.4 (13.3)	73.6 (13.2)	0.94
Abdominal surgery				
No		30 (68.2)	38 (77.6)	0.30
Yes		14 (31.8)	11 (22.4)	
Barthel index*				
≤30		31 (70.5)	37 (75.5)	0.83
35–60		6 (13.6)	5 (10.2)	
>60		7 (15.9)	7 (14.3)	
Median (25th, 75th percentile)		17.5 (0, 45)	10.0 (0, 32.5)	0.59
Severity of constipation^†^				
Group A		21 (47.7)	23 (46.9)	0.84
Group B		15 (34.1)	19 (38.8)	
Group C		8 (18.2)	7 (14.3)	
History of disease				
Hypertension		30 (68.2)	29 (59.2)	0.36
Cerebrovascular disease		22 (50.0)	29 (59.2)	0.37
Diabetes		15 (34.1)	17 (34.7)	0.95
Cardiovascular disease		12 (27.3)	16 (32.7)	0.57
Dementia		9 (20.5)	9 (18.4)	0.79
Anxiety/depression		7 (15.9)	12 (24.5)	0.30
Chronic lung disease		6 (13.6)	5 (10.2)	0.60
Peptic ulcer		4 (9.1)	7 (14.3)	0.43
Anemia		3 (6.8)	5 (10.2)	0.71
Parkinsonism		2 (4.5)	7 (14.3)	0.16
Malignancy except GI origin		2 (4.5)	3 (6.1)	0.99
Chronic hepatitis		2 (4.5)	3 (6.1)	0.99
Chronic kidney disease		1 (2.3)	3 (6.1)	0.61

*Barthel index [26] (0–100) is a scale used to measure performance in basic activities of daily living. A higher number is associated with a better performance.

^†^Participants were classified into three groups according to their bowel performance in the run-in period under the bowel routine protocol in long-term care:

group A: received enema more than once a week,

group B: received enema once a week or had less than three times of spontaneous bowel movements per week,

group C: 3–7 times of spontaneous bowel movements per week under usual care.

**Table 4:** Comparison of the treatment effect between CCH1 and lactulose.

Variables	Group, mean (95% CI)		
	CCH1 (*n* = 44)	Lactulose (*n* = 49)	Adjusted *P* ^‡^
Frequency of spontaneous bowel movement (SBM/wk)^§^			
Baseline (weeks −2~0)	2.7 (1.9, 3.4)	2.6 (2.0, 3.2)	0.907
Treatment (weeks 1~4)	6.9^†^ (6.1, 7.6)	4.5^†^ (3.5, 5.4)	<0.001
Treatment (weeks 5~8)	6.8^†^ (6.0, 7.7)	5.5^†^ (4.5, 6.6)	0.159
Follow-up (weeks 9~12)	3.7* (3.1, 4.4)	5.2^†^ (4.0, 6.4)	0.084
Frequency of rectal treatment (RT/wk)^||^			
Baseline (weeks −2~0)	1.4 (1.1, 1.8)	1.4 (1.1, 1.7)	0.958
Treatment (weeks 1~4)	0.5^†^ (0.4, 0.7)	0.9^†^ (0.7, 1.2)	0.030
Treatment (weeks 5~8)	0.3^†^ (0.2, 0.5)	0.6^†^ (0.4, 0.8)	0.051
Followup (weeks 9~12)	1.0* (0.8, 1.3)	0.8^†^ (0.5, 1.1)	0.561
Amount of rescue laxative use (MgO/wk)			
Baseline (weeks −2~0)	0 (0, 0)	0.4 (−0.1,0.8)	0.083
Treatment (weeks 1~4)	0.2 (−0.1, 0.5)	2.4* (1.1, 3.8)	0.005
Treatment (weeks 5~8)	1.7 (−0.4, 3.9)	10.4^†^ (6.1, 14.7)	0.002
Followup (weeks 9~12)	19.1^†^ (14.9, 23.2)	20.0^†^ (14.5, 25.4)	1.000
Laxative cost (USD/wk)^¶^			
Treatment (weeks 1~4)	1.4 (1.2, 1.5)	4.7 (4.2, 5.2)	<0.001
Treatment (weeks 5~8)	1.5 (1.3, 1.7)	5.0 (4.4, 5.7)	<0.001

Pairwise comparisons were performed for each treatment group with its baseline.

*P < 0.01; ^†^ *P* < 0.001.

^**‡**^ *P* value was adjusted by Bonferroni adjustment.

**^§^**SBM (spontaneous bowel movement) defined as stool passage without digital maneuver and without the use of suppository or enema on the same day.

**^||^**RT (rectal treatment) including enema, suppository use, or digital maneuver.

**^¶^**Laxative cost including the acquisition costs of study medication and rescue laxative.

**Table 5:** Comparison of stool consistency and stool amount between CCH1 (*n* = 44) and lactulose (*n* = 49) groups.

Variables	Group, mean (95% CI)		
	CCH1	Lactulose	Adjusted P*
Average score of bristol scale^†^			
Week −2–0	4.0 (3.8, 4.3)	4.2 (4.0, 4.3)	0.495
Week 1–4	4.7 (4.5, 4.9)	4.5 (4.3, 4.7)	0.475
Week 5–8	4.7 (4.5, 4.9)	4.8 (4.6, 5.0)	1.000
Week 9–12	4.5 (4.3, 4.8)	4.7 (4.5, 4.9)	1.000
Percentage of large stool amount^‡^			
Week −2–0	29.8 (21.5, 38.1)	32.9 (25.4, 40.3)	0.581
Week 1–4	36.6 (30.5, 42.8)	38.3 (30.8, 45.9)	1.000
Week 5–8	42.1 (34.9, 49.2)	38.7 (31.4, 46.0)	1.000
Week 9–12	37.0 (29.8, 44.3)	32.9 (25.2, 40.6)	1.000
Percentage of moderate stool amount^‡^			
Week −2–0	50.5 (42.0, 59.1)	52.9 (44.5, 61.2)	0.692
Week 1–4	52.2 (47.1, 57.4)	49.2 (41.0, 57.4)	1.000
Week 5–8	48.2 (41.9, 54.6)	47.5 (39.9, 55.0)	1.000
Week 9–12	53.2 (46.0, 60.4)	50.9 (42.9, 58.9)	1.000
Percentage of small stool amount^‡^			
Week −2–0	19.7 (13.1, 26.3)	14.2 (8.6, 19.9)	0.211
Week 1–4	11.1 (7.4, 14.8)	12.0 (7.5, 16.4)	1.000
Week 5–8	9.7 (5.6, 13.8)	13.8 (8.9, 18.7)	0.588
Week 9–12	9.8 (6.1, 13.5)	16.2 (11.0, 21.4)	0.142

*P value was adjusted by Bonferroni adjustment.

^†^Stool consistency was recorded by Bristol Stool Form Scale [25], ranging from 1 (separate hard lumps, like nuts) to 7 (watery, no solid pieces).

^‡^Stool amount was classified into three categories (small, <250 g; moderate, 250–500 g; large, >500 g) according to a national guidelines of the Registered Nurses Association [24].

**Table 6:** Global assessment of efficacy.^∗,†^

Variables	Group, number (%)		*P* Value
	CCH1 (*n* = 39)	Lactulose (*n* = 34)	
Marked improved	21 (53.8)	18 (52.9)	0.646
Slightly improved	12 (30.8)	9 (26.5)	
Unchanged	5 (12.8)	7 (20.6)	
Slightly worse	1 (2.6)	0 (0)	
Markedly worse	0 (0)	0 (0)	

*The global assessment of efficacy was evaluated at the end of treatment phase by patients themselves or their principal caregivers if their cognition was impaired.

^†^A total of 20 patients dropped out of the trial during the treatment phase.

**Table 7:** Compliance for study medication.*

Study medication	Group, mean (SD)		*P* Value
	CCH1	Lactulose	
Powder			
CCH1	97.1 (6.4)	—	0.772
CCH1 Placebo	—	97.4 (5.1)	
Syrup			
Lactulose	—	97.5 (5.1)	0.637
Lactulose Placebo	96.9 (6.4)	—	

*The experimental group received CCH1 powder and lactulose placebo. The active comparator group was given lactulose syrup and CCH1 placebo.

**Table 8:** Comparison of severe adverse events.

Variables		Group, number (%)	
		CCH1	Lactulose
Pneumonia		5 (11.4)	5 (10.2)
Upper GI bleeding		1 (2.3)	1 (2.0)
Acute gastroenteritis		0 (0)	2 (4.1)
Cellulitis		0 (0)	1 (2.0)
Urinary tract infection		0 (0)	2 (4.1)
Ileus		0 (0)	1 (2.0)

**Table 9:** Comparison of common adverse events (cumulative incidence >5%).

Variables	Group, number (%)		*P* Value*
	CCH1	Lactulose	
Flatulence	16 (36.4)	17 (34.7)	0.867
Albumin ↓	15 (34.1)	10 (20.4)	0.137
Diarrhea	15 (34.1)	17 (34.7)	0.951
Hiccup	13 (29.5)	12 (24.5)	0.583
Hemoglobin ↓	12 (27.3)	6 (12.2)	0.067
Abdominal pain	10 (22.7)	8 (16.3)	0.435
Bloating	9 (20.5)	11 (22.4)	0.815
Magnesium ↑	8 (18.2)	12 (24.5)	0.614
Acid regurgitation	8 (18.2)	8 (16.3)	0.813
URI	8 (18.2)	6 (12.2)	0.424
Red blood cell ↓	7 (15.9)	4 (8.2)	0.248
Sugar ↑	6 (13.6)	9 (18.4)	0.536
Calcium ↓	6 (13.6)	2 (4.1)	0.143
Sodium ↓	5 (11.4)	2 (4.1)	0.249
Alkaline phosphatase ↑	4 (9.1)	1 (2.0)	0.186
Albumin ↑	4 (9.1)	1 (2.0)	0.186
Potassium ↑	4 (9.1)	4 (8.2)	1.000
Phosphate ↑	4 (9.1)	1 (2.0)	0.186
Nausea	4 (9.1)	7 (14.3)	0.439
White blood cell ↓	3 (6.8)	2 (4.1)	0.665
Sugar ↓	3 (6.8)	2 (4.1)	0.665
Blood urea nitrogen ↑	3 (6.8)	5 (10.2)	0.718
Dizziness	3 (6.8)	1 (2.0)	0.341
Cough	3 (6.8)	4 (8.2)	0.561
Skin rash	2 (4.5)	3 (6.1)	1.000
Platelet ↑	2 (4.5)	3 (6.1)	1.000
Aspartate aminotransferase ↑	2 (4.5)	4 (8.2)	0.680
Alanine aminotransferase ↑	2 (4.5)	3 (6.1)	1.000
Creatinine ↑	2 (4.5)	3 (6.1)	1.000
Triglyceride ↑	2 (4.5)	5 (10.2)	0.440
Vomiting	2 (4.5)	3 (6.1)	1.000
Diaper rash	1 (2.3)	5 (10.2)	0.207
Cholesterol ↑	1 (2.3)	4 (8.2)	0.365
Cholesterol ↓	1 (2.3)	3 (6.1)	0.619
Creatine phosphokinase ↑	0 (0)	3 (6.1)	0.244
Uric acid ↑	0 (0)	3 (6.1)	0.244
Pruritus	0 (0)	3 (6.1)	0.244
Chest tightness	0 (0)	3 (6.1)	0.244

*A *χ* ^2^ test or Fisher exact test where appropriate was used to compare the number of patients with adverse events between two groups.
